# The Transcriptomic Landscape of Gastric Cancer: Insights into Epstein-Barr Virus Infected and Microsatellite Unstable Tumors

**DOI:** 10.3390/ijms19072079

**Published:** 2018-07-17

**Authors:** Irene Gullo, Joana Carvalho, Diana Martins, Diana Lemos, Ana Rita Monteiro, Marta Ferreira, Kakoli Das, Patrick Tan, Carla Oliveira, Fátima Carneiro, Patrícia Oliveira

**Affiliations:** 1 Department of Pathology, Centro Hospitalar de São João, Porto 4200-319, Portugal; irene.gullo12@gmail.com (I.G.); fcarneiro@ipatimup.pt (F.C.); 2 Department of Pathology, Faculty of Medicine of the University of Porto (FMUP), Porto 4200-319, Portugal; 3 Institute of Molecular Pathology and Immunology, University of Porto (Ipatimup), Porto 4200-135, Portugal; jcarvalho@ipatimup.pt (J.C.); dianam@ipatimup.pt (D.M.); dlemos@ipatimup.pt (D.L.); anaritapatriciomonteiro@gmail.com (A.R.M.); martaf@ipatimup.pt (M.F.); carlaol@ipatimup.pt (C.O.); 4 Instituto de Investigação e Inovação em Saúde (i3S), University of Porto, Porto 4200-135, Portugal; 5 Cancer and Stem Cell Biology Program, Duke-NUS Medical School, Singapore 169857, Singapore; kakoli.das@duke-nus.edu.sg (K.D.); gmstanp@duke-nus.edu.sg (P.T.); 6 Genome Institute of Singapore, Biopolis, Singapore 138672, Singapore; 7 Cancer Science Institute of Singapore, National University of Singapore, Singapore 117599, Singapore

**Keywords:** gastric cancer, transcriptomic profiling, Epstein-Barr Virus, EBV, microsatellite instability, MSI

## Abstract

Background: Epstein-Barr Virus (EBV) positive and microsatellite unstable (MSI-high) gastric cancer (GC) are molecular subgroups with distinctive molecular profiles. We explored the transcriptomic differences between EBV+ and MSI-high GCs, and the expression of current GC immunotherapy targets such as PD-1, PD-L1, CTLA4 and Dies1/VISTA. Methods: Using Nanostring Technology and comparative bioinformatics, we analyzed the expression of 499 genes in 46 GCs, classified either as EBV positive (EBER in situ hybridization) or MSI-high (PCR/fragment analysis). PD-L1 protein expression was assessed by immunohistochemistry. Results: From the 46 GCs, 27 tested MSI-high/EBV−, 15 tested MSS/EBV+ and four tested MSS/EBV−. The Nanostring CodeSet could segregate GCs according to MSI and, to a lesser extent, EBV status. Functional annotation of differentially expressed genes associated MSI-high/EBV− GCs with mitotic activity and MSS/EBV+ GCs with immune response. PD-L1 protein expression, evaluated in stromal immune cells, was lower in MSI-high/EBV− GCs. High mRNA expression of *PD-1*, *CTLA4* and *Dies1/VISTA* and distinctive *PD-1/PD-L1* co-expression patterns (*PD-1*^high^/*PD-L1*^low^, *PD-1*^high^/*PDL1*^high^) were associated with MSS/EBV+ molecular subtype and gastric cancer with lymphoid stroma (GCLS) morphological features. Conclusions: EBV+ and MSI-high GCs present distinct transcriptomic profiles. GCLS/EBV+ cases frequently present co-expression of multiple immunotherapy targets, a finding with putative therapeutic implications.

## 1. Introduction

Gastric Cancer (GC) is a heterogeneous disease at the morphological and molecular levels [1]. Numerous somatic gene mutations, copy-number variations, translocations/inversions, as well as epigenetic and transcriptional changes have been described so far in this disease [2]. However, very few prognostic and predictive biomarkers of therapy response have been introduced into clinical practice [3], and the “one-size-fits-all” is currently the main approach to treat GC patients. Understanding GC molecular heterogeneity and deciphering its players is urgently needed to define more homogenous and targetable biological subtypes.

Recently, several groups [4,5,6] were able to uncover distinct molecular subtypes with potential clinical significance and therapeutic implications, through the integrative analysis of large-scale genomic and proteomic data. The landmark study of GC that attempted to determine a molecular-based stratification was carried out by The Cancer Genome Atlas (TCGA) research network [4]. By investigating exome sequences, copy-number alterations, DNA methylation, gene expression and proteomic data, TCGA classified GC into four subtypes: (1) Epstein-Barr Virus positive (EBV+) GCs, (2) GCs with microsatellite instability (MSI-high), (3) genomically stable GCs and (4) GCs with chromosomal instability.

The current study focuses on the EBV+ and MSI-high molecular subtypes. The TCGA study has shown that EBV+ GCs display distinctive molecular characteristics: high genomic-wide hypermethylation with *CDKN2A* silencing, amplification of *JAK2*, *CD274*, *PDCD1LG2*, and *ERBB2*, mutations of *PIK3CA*, *ARID1A* and *BCOR*, and very rarely *TP53* mutations. Most EBV+ GCs were in the proximal stomach (fundus/body), affecting mainly male patients and had a better prognosis than other subtypes [4,6]. MSI-high GCs are characterized by: a hypermethylation phenotype associated with *MLH1* silencing, and accordingly a hypermutated status often targeting *TP53*, *KRAS*, *ARID1A*, *PIK3CA*, *ERBB3*, *PTEN* and *HLA-B* [4]. Patients with MSI-high tumors are generally diagnosed at older age, are mainly females and display better prognosis than patients with the genomically stable subtype, but worse than EBV+ GC patients [4,5,6,7].

Based on gene expression analysis, the TCGA study revealed four clusters of differentially expressed (DE)-genes, which have to some extent correspondence with specific molecular subtypes [4], thus demonstrating the robustness of the proposed classification. An mRNA cluster enriched in genes involved in mitotic pathways was associated with the MSI-high subtype, while for EBV+ GCs an enrichment in genes associated with immune signaling was observed [4]. In-depth studies based on TCGA transcriptomic data confirmed that genes related to T-cell cytotoxic function, pro-inflammatory cytokines signaling and interferon gamma (IFNγ) response were highly expressed in EBV+ GCs and, to a lesser extent, in MSI-high GCs [2,6,8,9]. Moreover, PD-1/PD-L1 mRNA and protein expression are frequently present in both EBV+ and MSI-high molecular subtypes [8,9]. Accordingly, EBV+ and MSI-high GCs are frequently characterized by prominent immune infiltrate and may display the morphological features of GC with lymphoid stroma (GCLS) [9,10,11,12,13], a rare histological phenotype characterized by prominent lymphoid infiltration [14].

These molecular data offer a rationale to investigate the value of EBV and MSI molecular status in predicting the efficacy of immunotherapy in GC. Currently, the only predictive biomarkers used to select GC patients for targeted immunotherapy, i.e., Pembrolizumab (anti-PD-1 antibody) [15,16,17] are MSI-high status and/or the expression of PD-L1 by immunohistochemistry (IHC) in cancer epithelial cells and/or in immune cells of the tumor microenvironment. However, several clinical trials have shown that GC patients benefit from PD-1/PD-L1 immune checkpoint inhibitors regardless of PD-L1 expression [18,19,20]. Therefore, the investigation of morphological and new molecular profiles in this context might help optimizing treatment selection. A recent clinical trial with Pembrolizumab across different cancer types, including GC, demonstrated that tumors with an expression signature enriched in genes related to cytotoxic effector signaling, pro-inflammatory cytokines/chemokines and IFNγ response, showed a T-cell inflamed phenotype associated with better response to targeted immunotherapies [21]. These and other recent evidences suggest that measuring multiple immunological determinants may be relevant to predict who will respond to targeted immunotherapies [22]. Therefore, in this context, the presence of EBV positivity and MSI-high status in GC may serve to select patients for immune checkpoint inhibitor therapy [8,9,13].

Although a growing number of publications have focused on the study of EBV+ and MSI-high molecular subtypes separately or as a group, an in-depth analysis of the contribution of EBV infection and MSI status to the transcriptomic landscape of GC is still lacking. In this study, we performed an unbiased analysis, aimed at uncovering differences within the gene expression profiles of EBV+ and MSI-high molecular subtypes, and at analyzing the expression profile of current targets for immunotherapy in GC.

## 2. Results

**Figure 1:**
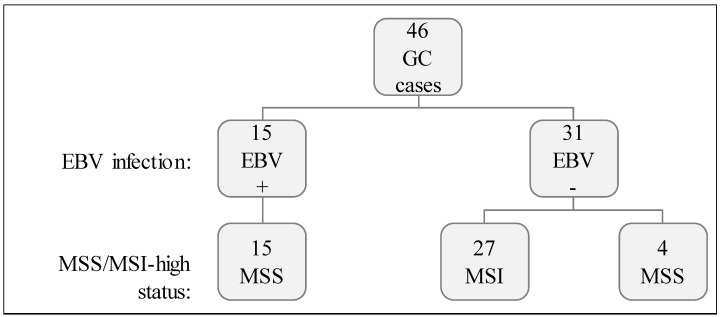
Cohort characterization for EBV infection and MSI status.

We studied 46 GCs characterized for two molecular features: (1) EBV infection and, (2) MSI-high status (Figure 1). Fifteen out of 46 GC (32.6%) displayed EBV positivity (EBV+) and the remaining 31/46 (67.4%) were negative (EBV−). Concerning MSI status, 27/46 (58.7%) were MSI-high while 19/46 (41.3%) were microsatellite stable (MSS). All EBV+ GC cases were MSS, while most (27/31) EBV− cases were MSI-high (Figure 1).

### 2.1. EBV+ and MSI-High GCs Displayed Distinct Transcriptomic Signatures

**Figure 2:**
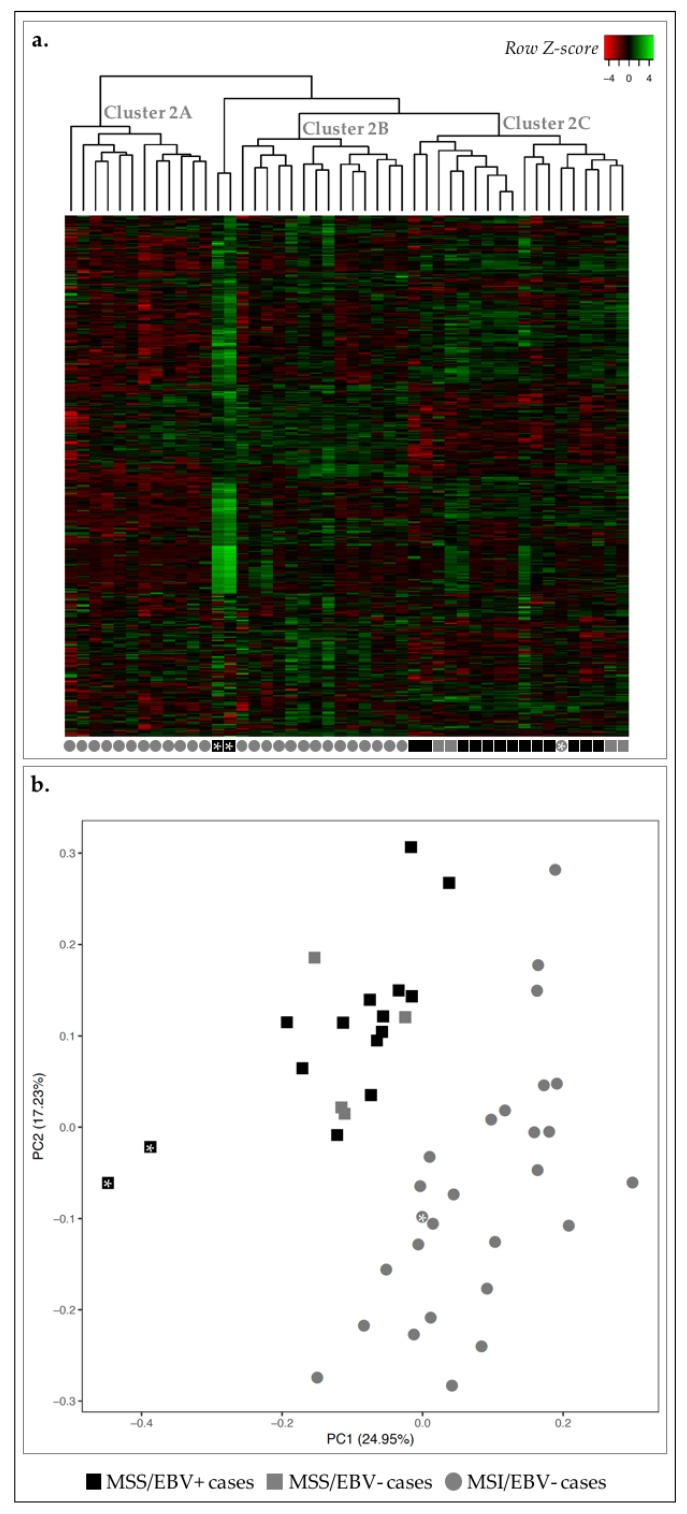
Transcription profile of 46 GC cases for 499 genes in the Nanostring CodeSet. Asterisks are used for cross-reference between the two figures and as links to the main text. (**a**) Heatmap for the expression of all genes in all GC cases (log 2 and *Z*-score scaled). Indicated are three main clusters: 2A–C. (**b**) PCA for principal components 1 and 2 for the 46 GC samples. Black squares correspond to MSS/EBV+ cases (*n* = 15), gray squares to MSS/EBV− cases (*n* = 4), gray circles to MSI-high/EBV− cases (*n* = 27).

We analyzed the transcriptomic landscape of the 46 GC cases, aimed at unveiling differences between EBV+ and MSI-high GC subtypes. For this, we have used a previously published Nanostring nCounter CodeSet [23], which comprised 499 genes associated with oncogenic signaling pathways, GC molecular subtype signatures and immune response. After adequate data analysis and normalization, the expression of the 499 genes was plotted in a heatmap and non-hierarchical clustering and principal component analysis (PCA) were performed (Figure 2).

Concerning MSI-high status, we observed that 26/27 MSI-high cases were clustered in clusters 2A and 2B, and 17/19 MSS cases were clustered together in cluster 2C (Figure 2a). Using a PCA, we observed a clear separation between MSI-high and MSS cases (circles vs. squares, Figure 2b). Concerning EBV infection, we observed that most EBV+ cases (13/15) were clustered together in cluster 2C, with the remaining two EBV+ cases found within/close to cluster 2B (Figure 2a). With the PCA, almost all EBV+ cases were separated from EBV− cases (black vs. gray, Figure 2b).

As we correlated the information from the heatmap and dendrogram and the PCA, we could observe the clear separation of two MSS/EBV+ cases in the PCA, which likely reflects overall higher expression in these two cases (black squares with asterisk, Figure 2). Furthermore, we observed the MSI-high/EBV− case in MSS-rich cluster 2C, however clustered among MSI-high cases in the PCA, highlighting the differences in the clustering strategies (gray circle with asterisk, Figure 2). These results suggested that the transcriptomic landscape assessed can clearly distinguish GC cases with an MSS phenotype from those with MSI-high phenotype, and, to a lesser degree, EBV+ from EBV− cases.

To further reinforce these findings, we used the partitioning method *k-means* and several values of *k* to understand how strongly the gene expression profile followed the classification MSI-high or EBV+. The best results were observed for a value of *k* = 3, as the clusters calculated were the most homogeneous in terms of molecular subtypes, particularly for the MSS/MSI-high status. In fact, all MSI-high cases fell into cluster I and were perfectly separated from MSS cases that fell into clusters II and III (Table 1). For EBV− cases, the clustering was more heterogeneous, spreading across two different clusters (I and III). Of notice, if we disregard the two samples in cluster II, which correspond to those previously shown to display an abnormally high global expression profile (black squares with asterisk, Figure 2), homogeneity in cluster III becomes evident.

Our observations show that the gene expression profile assessed with the Nanostring CodeSet was sufficient to illustrate the molecular separation of MSS and MSI-high molecular subtypes. This analysis also provides the *rationale* to derive a smaller specific gene expression signature that strongly discriminates MSS and MSI-high phenotypes and, to a lesser extent, EBV infection status. To better understand this, we next studied each molecular subtype independently, aiming at uncovering the biological meaning of the gene expression profiles associated with each phenotype.

### 2.2. MSI-High GC Cases Displayed a Mitotic Signature, While MSS GC Cases Showed an Immune Response Signature

**Figure 3:**
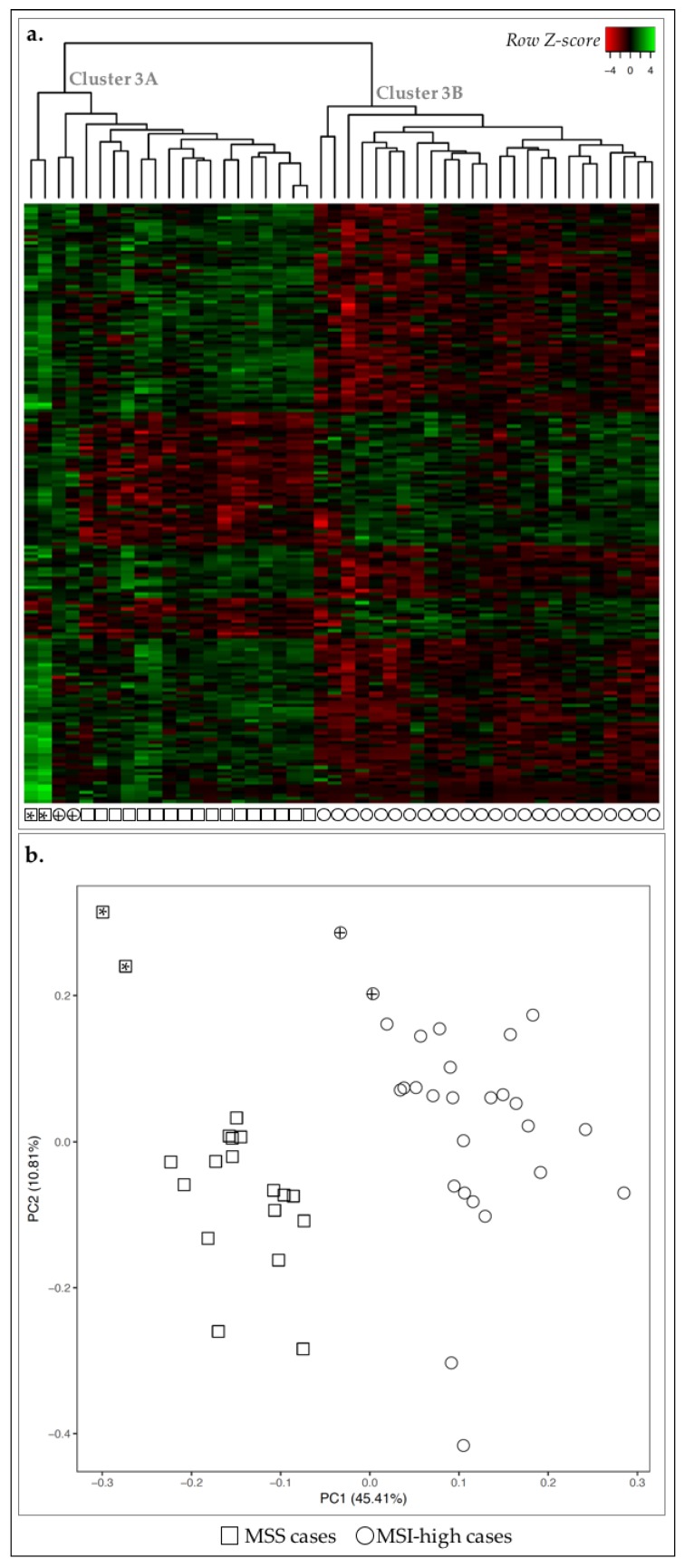
Expression profile of the 46 GC cases for 193 DE-genes between MSS and MSI-high cases. Asterisks and crosses are used for cross-reference between the two figures and as links to the main text. (**a**) Heatmap for the expression of the 193 DE-genes (log 2 and *Z*-score scaled). Indicated are two main clusters: 3A and 3B. (**b**) PCA for principal components 1 and 2 for the 46 GC samples. Squares correspond to MSS cases (*n* = 19) and circles to MSI-high (*n* = 27). Circles with cross or squares with asterisk for correspondence between panels (**a**) and (**b**) and described in the main text.

We first compared the transcriptomic landscape of MSS and MSI-high cases and detected 193 genes DE-genes (False Discovery Rate (FDR) ≤ 0.05 and 1.5 ≤ fold-change ≤ 0.6). By performing a non-hierarchical clustering using specifically these DE-genes, we observed that all MSS cases (*n* = 19/19) and the majority of MSI-high cases (*n* = 25/27), were clustered together (cluster 3A and cluster 3B, respectively) (Figure 3a), while two MSI-high outlier cases clustered together with the MSS cases (cluster 3A, circles with cross, Figure 3a). However, these two cases were clustered closer to the remaining MSI-high cases than MSS with PCA, with over 50% of data variability considered (Figure 3b). In fact, PCA exhibited a clear separation of cases according to MSS/MSI-high status.

We next searched for biological annotations among the 193 DE-genes between MSS and MSI-high cases [24,25]. We observed enrichment in the more generalist terms such as signal peptide and disulfide bond, as well as in regulation of cell proliferation and chemotaxis (Table 2).

From the 193 DE-genes, 55 were upregulated in MSI-high cases and annotated to terms associated with cell division. The remaining 138 DE-genes, downregulated in MSI-high cases, were related to chemotaxis and immune response. The 55 upregulated DE-genes in MSI-high cases were significantly associated with three annotation clusters: cluster 1 associated with cell cycle and cell division; cluster 2 with cytoskeleton and; cluster 3 associating more specific terms such as ‘mitotic spindle organization’ (Table 3). For the 138 downregulated DE-genes, eight clusters were significantly enriched associated with the terms: signal peptide and disulfide bond; chemotaxis; leukocyte and lymphocyte activation; chemokine activity; response to stimuli; regulation of cell death; cell migration and motility, and; polysaccharide binding (Table 3). Taken together, these results showed that MSI-high cases likely present increased cell division and decreased immune response and cell migration.

### 2.3. EBV+ GC Cases Were Associated with Immune Response Signature

**Figure 4:**
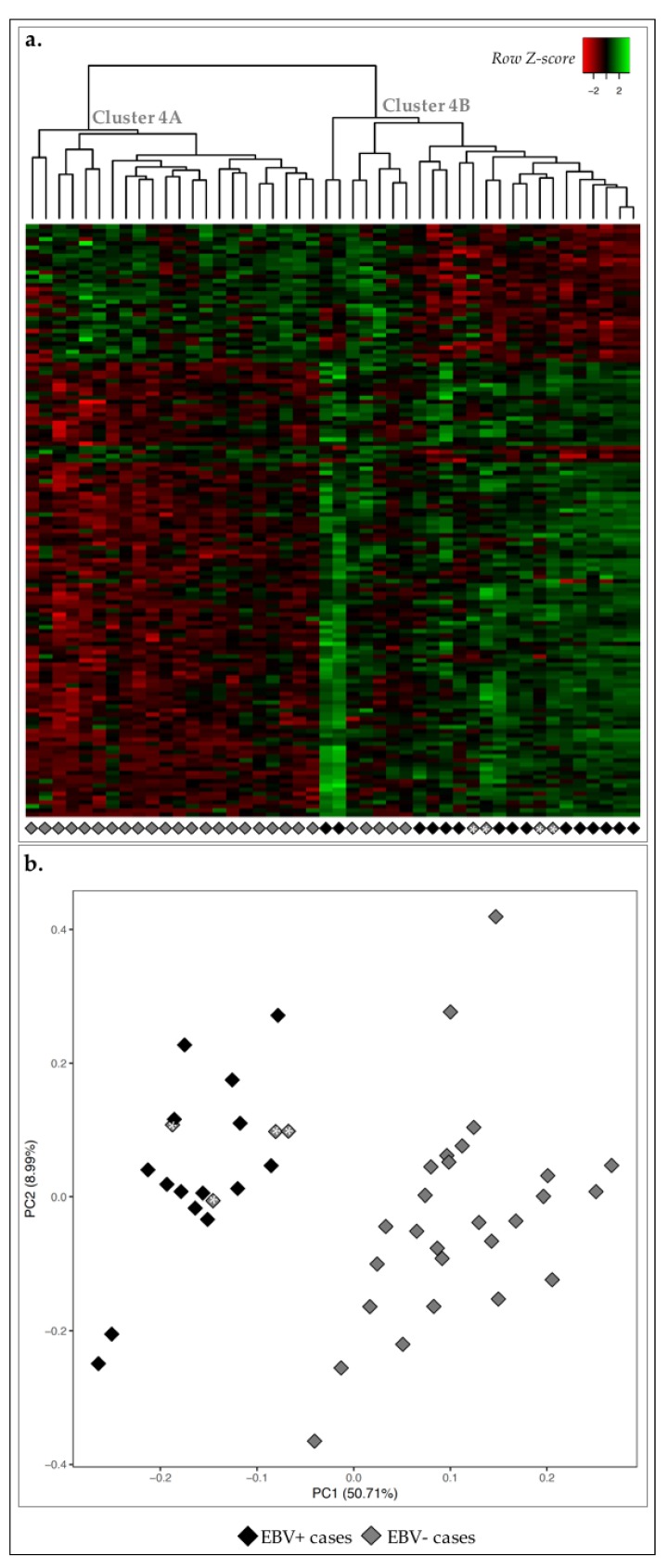
Expression profile of the 46 GC cases for 142 DE-genes between EBV positive and negative cases. (**a**) Heatmap for the expression of the 142 DE-genes (log 2 and *Z*-score scaled). Indicated are two main clusters: A and B. (**b**) PCA for principal components 1 and 2 for the 46 GC samples. Black diamonds correspond to EBV+ cases (*n* = 15) and white diamonds to EBV− cases (*n* = 31). Diamonds with asterisk for correspondence between panels (**a**) and (**b**) and described in the main text.

Next, we compared the transcriptomic landscape of EBV+ and EBV− GC cases. We detected 142 DE-genes and non-hierarchical clustering revealed two major clusters: cluster 4A with 22/31 cases EBV− and; cluster 4B constituted by all 15 EBV+ cases plus the remaining 9 EBV− cases (Figure 4a). This separation between EBV+ and EBV− cases was partially recapitulated by PCA: 4/9 EBV− cases clustered together with EBV+ cases in the non-hierarchical clustering, were similarly grouped with PCA (gray diamonds with asterisk, Figure 4). This showed an overall less homogenous clustering according to EBV status, demonstrating that the MSI-high status was a stronger marker in GC.

We then performed biological annotation of the 142 DE-genes and observed a significant enrichment in terms such as chemotaxis and immune response (Table 4).

From the 142 DE-genes, 105 were upregulated EBV+ cases vs. EBV− cases and belonged to six enriched clusters: signal peptide and disulfide bond; chemotaxis and motility; leukocyte and T-cell activation; chemokines and chemokine activity; immune system development; and T-cell differentiation (Table 5). The 37 genes downregulated in this comparison were enriched in two clusters: cell division, mitosis, and cell cycle (Table 5).

Our results show that EBV infection is not as determinant as MSI-high status. Nevertheless, the presence of this virus in GC samples was associated with an immune T-cell inflamed phenotype, in line with current literature [4,6,8].

### 2.4. MSS/MSI Phenotype Classification Was the Major Molecular Classifier in GC

Given that MSI and EBV status were part of the molecular classification for GC proposed by TCGA [4], we next assessed the differential expression profile of our GC cases taking into account both molecular classifications. We detected 166 DE-genes associated with biological annotations such as interleukin-8-like chemokine, signal peptide and chemotaxis (Table 6).

**Figure 5:**
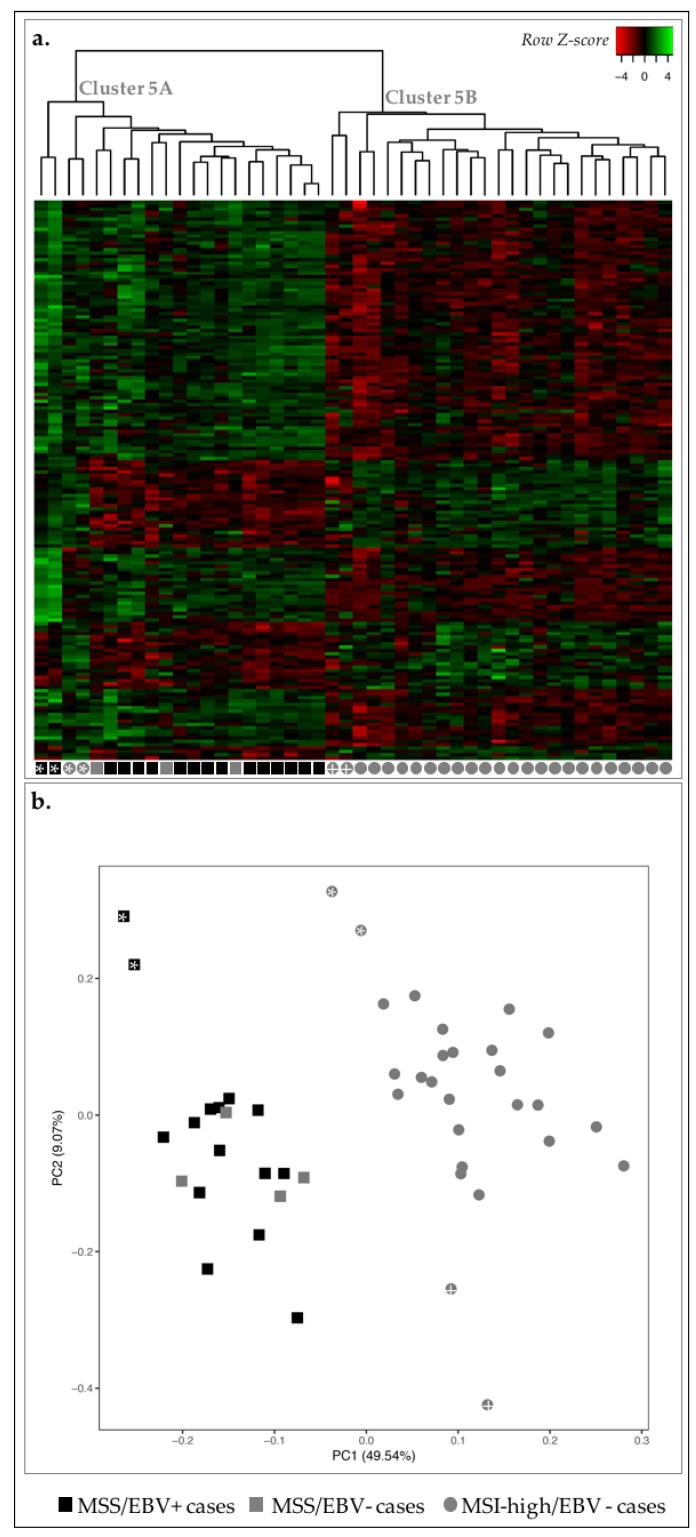
Expression profile of the 46 GC cases for 166 DE-genes between MSS/EBV+ and MSI-high/EBV− cases. (**a**) Heatmap for the expression of the 166 DE-genes (log 2 and *Z*-score scaled). Indicated are two main clusters: A and B. (**b**) PCA for principal components 1 and 2 for the 46 GC samples. Black squares correspond to MSS/EBV+ cases (*n* = 15), gray squares to MSS/EBV− cases (*n* = 4) and gray circles to MSI-high/EBV− cases (*n* = 27).

From the 166 DE-genes, 117 were upregulated and 49 downregulated DE-genes in MSS/EBV+ cases vs. MSI-high/EBV−genes. Next, we plotted the expression of these DE-genes for all 46 GC cases and observed that, despite adding both molecular classifiers, MSI-high and MSS cases remained well separated (Figure 5a). As observed before, two MSI-high/EBV− GC cases were clustered together with MSS cases (*cluster 5A*, Figure 5a, gray circles with asterisk and *cluster 3A*, Figure 3a, circles with cross). Nevertheless, with PCA, the same two cases were clustered closer to MSI-high/EBV− GC cases. PCA separated two other MSI-high/EBV− cases, which were already loosely clustered in cluster 5B (Figure 5, gray circles with cross).

Separate annotation of up- and downregulated DE-genes in MSS/EBV+ cases revealed significant enrichment for five annotation clusters for upregulated genes: signal peptide and disulfide bond; chemotaxis and locomotion; leukocyte and T-cell activation; chemokines and chemokine activity, and; actin fibers (Table 7). Downregulated genes were separated in two clusters: cell cycle and mitosis, and cytoskeleton.

These results were comparable to those previously observed when considering the molecular classifiers independently. To understand whether this was due to common set of DE-genes across analyses, we next compared the DE-genes obtained for each comparison: MSS with MSI-high cases; EBV+ with EBV− cases and; MSS/EBV+ with MSI-high/EBV− cases.

**Figure 6:**
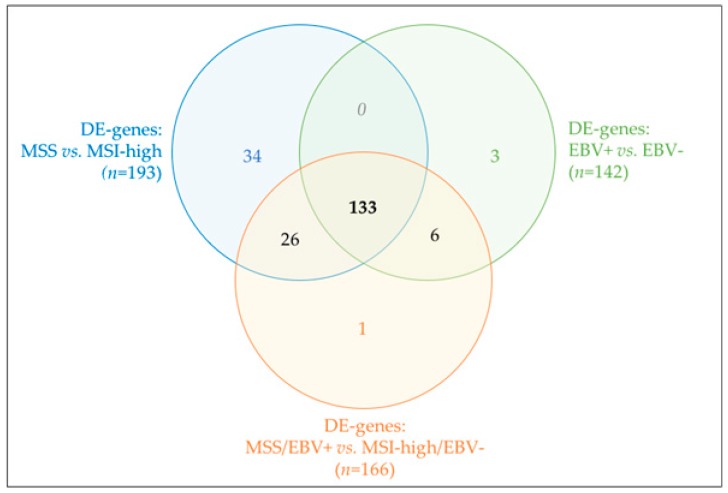
Venn diagram for shared (or not) DE-genes across the three analyses performed.

Most DE-genes were shared by the three analyses (*n* = 133, Figure 6), thus justifying the similar biological annotation enrichments obtained. Unlike EBV−-based classification, many DE-genes derived specifically from the MSI-high/MSS-based classification and became lost when combining both molecular subtypes (*n* = 34, Figure 6). Functional enrichment of this particular set of DE-genes, although without any FDR-significant results, pointed toward enrichment in extracellular matrix terms. Altogether, our results pinpointed MSI-high/MSS phenotype as the major molecular classifier in our GC cohort, independently of EBV− *tatus* classification.

### 2.5. PD-L1 and PD-1 Displayed Opposite mRNA Expression Patterns and Were Differently Associated with GC Molecular Subtypes and Morphological Features

**Figure 7:**
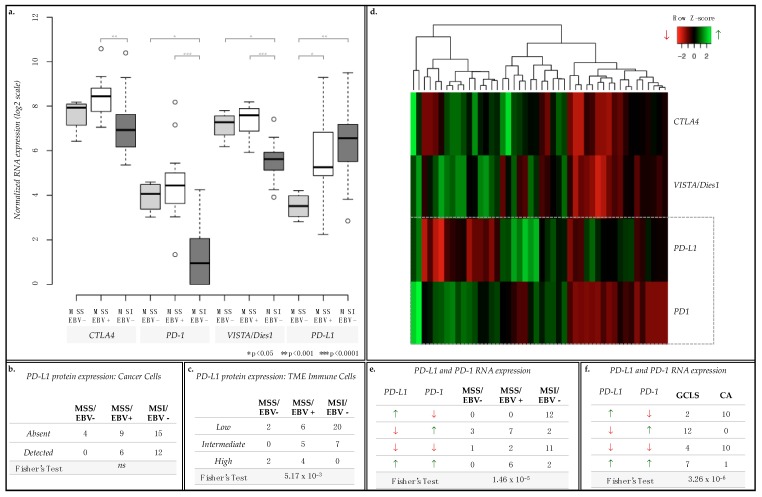
mRNA expression of immune checkpoint regulators *CTLA4*, *PD-1*, *VISTA/Dies1* and *PD-L1* and protein expression of PD-L1. (**a**) Boxplot for the normalized mRNA expression in log2 scale of *CTLA4*, *PD-1*, *VISTA/Dies1* and *PD-L1*. (**b**,**c**) Contingency tables for PD-L1 protein expression evaluated by in cancer cells (**b**, absent or detected) and in immune cells of the TME (**c**, low, intermediate, or high expression level). (**d**) Heatmap for the mRNA expression of each of the immune checkpoints assessed per case. (**e**,**f**) Contingency tables for combined *PD-L1* and *PD-1* mRNA expression for GC cases separated by MSI status and EBV infection (**e**) or by morphological characteristics (gastric cancer with lymphoid stroma, GCLS, or conventional-type adenocarcinoma, CA) (**f**). Green upward arrows for higher mRNA expression and red downward arrows for lower mRNA expression, as presented in the heatmap.

In GC, several clinical trials have been targeting immune checkpoint regulators, such as CTLA4, PD-1, PDL1 and VISTA/Dies1 [26]. Given the observed associations between immune response terms and MSS/EBV+ cases, we further assessed the mRNA expression of these immune checkpoint regulators across all cases from the 3 GC groups represented in our series (15 MSS/EBV+, 4 MSS/EBV− and 27 MSI-high/EBV−, Figure 7). *CTLA4*, *PD-1* and *VISTA/Dies1*, but not *PD-L1* mRNA expression was significantly enriched in MSS/EBV+ cases (Figure 7a). Therefore, we analyzed PD-L1 protein expression in cancer cells and in the immune cells infiltrating the tumor microenvironment (TME) to understand this difference. In cancer epithelial cells, PD-L1 protein expression did not differ between GC groups (Figure 7b). However, in the immune cells of the TME, MSI-high/EBV− cases often presented low expression of PD-L1, while MSS/EBV+ showed variable PD-L1 expression across all categories, from low to high (Figure 7c, Fisher’s Exact Test *p*-value = 7.71 × 10^−3^)

These results prompted us to re-analyze the mRNA expression of the four immune checkpoint regulators on a case-by-case manner. While *CTLA4* and *VISTA/Dies1* followed the expression pattern of *PD-1*, *PD-L1* varied in an inverse manner in a large set of cases: for example, GC cases with highest expression of *PD-L1* displayed the lowest expression of *PD-1* (Figure 7d).

To validate this observation, we assessed the number of GC cases for each of the four PD-L1/PD-1 co-expression scenarios observed: (1) high expression of *PD-L1* and low expression of *PD-1* (*PD-L1*^high^/*PD-1*^low^, *n* = 12); (2) low expression of *PD-L1* and high expression of *PD-1* (*PD-L1*^low^/*PD-1*^high^, *n* = 12); (3) low expression for both (*PD-L1*^low^/*PD-1*^low^, *n* = 14) and; (4) high expression for both (*PD-L1*^high^/*PD-1*^high^, *n* = 8, Figure 7d,e). We observed that most MSS/EBV+ cases were either *PD-L1*^low^/*PD-1*^high^ or *PD-L1*^high^/*PD-1*^high^ (*n* = 7 and 6, respectively, Figure 7e), while most MSI-high/EBV− cases were *PD-L1*^high^/*PD-1*^low^ or *PD-L1*^low^/*PD-1*^low^ (*n* = 23, Figure 7e) (Fisher’s Exact test, *p* = 1.46 × 10^−5^).

These significant results led us to further characterize our GC cohort for morphological characteristics by histopathological analysis. As a significant fraction of cases displayed a prominent lymphoid infiltration in the tumor stroma, showing the morphological features of GCLS, we stratified the GC series into GCLSs (*n* = 25) and conventional-type adenocarcinomas (CA), i.e., GC cases not presenting the morphological features of GCLS (*n* = 21). While most GCLS cases presented a *PD-L1*^low^/*PD-1*^high^ or *PD-L1*^high^/*PD-1*^high^ (*n* = 12 + 7, respectively, Figure 7f), CA cases either displayed a *PD-L1*^high^/*PD-1*^low^ or *PD-L1*^low^/*PD-1*^low^ (*n* = 10 + 10, respectively, Figure 7f). By combining this morphological characterization with the previously described molecular subtypes (Table 8), we observed that: (1) 20/21 MSI-high/EBV− CA cases presented a *PD-L1*^high^/*PD-1*^low^ or a *PD-L1*^low^/*PD-1*^low^ expression pattern; (2) 13/15 MSS/EBV+/GCLS cases presented either a *PD-L1*^low^/*PD-1*^high^ or a *PD-L1*^high^/*PD-1*^high^ expression pattern; (3) 3/4 MSS/EBV−/GCLS cases presented a *PD-L1*^low^/*PD-1*^high^ co-expression pattern.

**Figure 8:**
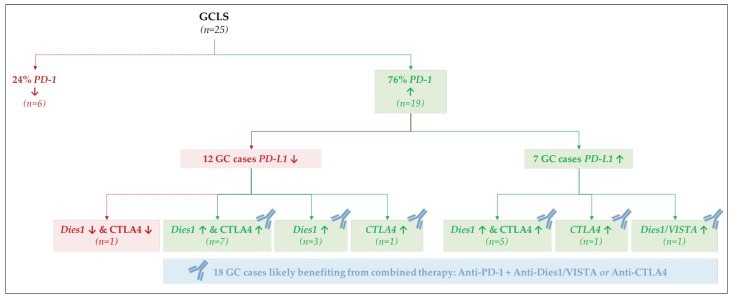
mRNA co-expression patterns for *PD-1*, *PD-L1*, *Dies1/VISTA* and *CTLA4* in GCLS cases.

The most important observation was that 19/25 GCLS cases displayed high *PD-1* mRNA expression, independently of *PD-L1* expression (12/19—low *PD-L1;* 7/19—high PD-L1). We then analyzed the expression of the other GC immunotherapy targets in the subset of GCLS: *Dies1/VISTA* and *CTLA4*. From the 12 GCLS cases with high *PD-1* and low *PD-L1* mRNA expression, 10 displayed high *Dies1/VISTA* mRNA expression (*n* = 7 + 3, Figure 8) and 8 high *CTLA4* mRNA expression (*n* = 7 + 1, Figure 8). From the remaining seven GCLS cases with high *PD-1* and *PD-L1* mRNA expression, all displayed high *Dies1/VISTA* and/or *CTLA4* mRNA expression (Figure 8).

Altogether, this gene-oriented analysis showed that *PD-L1* and *PD-1* exhibit particular co-expression patterns in an MSS/MSI-high and EBV infection-dependent manner. Moreover, our results suggest that the evaluation of the tumor immune infiltrate by histopathological analysis strengthened the stratification of GC cases and helped identifying more homogenous biological subgroups in terms of co-expression of immune checkpoint regulators.

## 3. Discussion

In this study, we explored the transcriptomic profile of EBV+ and MSI-high GC, using a Nanostring CodeSet with 499 genes involved in oncogenic signaling, immune response and molecular gene expression signatures. This small gene expression panel could segregate GCs of our cohort according to MSI-high status and, to a lesser extent, EBV infection, and was sufficient to reproduce the taxonomy developed by TCGA.

EBV infection and MSI-high status represent two alternative pathways of gastric carcinogenesis and two mutually exclusive GC molecular subtypes [11,12,27]. Herein, we confirmed that all EBV+ cases showed an MSS phenotype, and vice versa, that all MSI-high cases were negative for EBV infection.

When we focused on determining clusters of biologically-related annotation terms, underlying the DE-genes found for the two GC molecular subtypes, we found that MSI-high tumors showed an enrichment in genes related to DNA replication and mitotic cell cycle, as previously reported [4,5]. MSI-high status leads to the accumulation of numerous frameshift mutations throughout the genome [28] and may determine the inactivation of key tumor suppressor genes, including those involved in DNA damage repair, cell cycle control and apoptotic signaling [29]. Accordingly, as demonstrated in the MSI-high colorectal cancer model [30], mutations providing proliferative and survival advantage are selected during MSI-high GC initiation and/or progression, conferring a proliferative state. In contrast, EBV+ tumors showed a downregulation of genes involved in mitotic pathways.

By functional annotation of genes discriminating EBV+ tumors, we identified a gene signature involved in immune pathways, confirming the data already reported in the literature [4,6,8,31,32]. The immune signature was enriched for genes related to T-cell differentiation, cytotoxic signaling, pro-inflammatory cytokines/chemokines, leukocyte migration and genes of the immune checkpoint inhibitors pathways. These features reflect the immunogenicity of EBV infection and provide evidence of the biological significance of immune cell infiltration in EBV+ tumors [33]. Accordingly, the DE-genes downregulated in MSI-high cases, hence upregulated in MSS cases, were also found to be associated with immune response and cell migration. Therefore, in this study we demonstrated, using unbiased bioinformatics analyses, that the transcriptomic landscape of GCs with EBV+ and MSI-high phenotypes is different, associating each molecular entity with enrichment of different biological terms, i.e., mitotic activity and immune response. In this study, gene expression analysis was performed through the Nanostring Technology Platform, which has shown excellent robustness and sensitivity for the analysis of formalin-fixed paraffin-embedded (FFPE) samples [34]. Moreover, several authors have shown the reproducibility of the results obtained in GC tissues through Nanostring technology, using distinct molecular platforms [23,34]. Importantly, our results were able to confirm the transcriptomic data obtained in TCGA study, thus further contributing for the validation of the Nanostring CodeSet. In future studies, it would be interesting to confirm the enrichment of mitotic pathways in MSI GC cohorts, by investigating mitotic activity/index through histopathological analysis, as demonstrated already in the colorectal cancer model [30,35].

We also investigated the expression of molecules involved in immune checkpoint inhibitors pathways and current targets for immunotherapy in GC [26]. By assessing PD-L1 protein expression by IHC, the current predictive biomarker used for selecting GC patients eligible for Pembrolizumab immunotherapy [16], we found that PD-L1 protein expression, evaluated in cancer cells, showed no significant differences between the two molecular subgroups. However, when we evaluated PD-L1 expression in immune cells of the TME, most MSI-high/EBV− cases presented low expression, when compared to MSS/EBV+ tumors. This result shows the value of evaluating protein expression in tissue sections to improve knowledge of topographic distribution of molecular markers.

We also analyzed *PD-1*, *CTLA4* and *Dies1/VISTA* mRNA expression and observed that all were significantly enriched in MSS/EBV+ cases, in comparison with MSI-high/EBV− cases. This result highlighted the high correlation of *PD-1*, *CTLA4* and *Dies1/VISTA* increased mRNA expression with EBV+ cases, but not with EBV− cases. However, as we analyzed *PD-L1* mRNA expression, we did not observe any significant difference between the two molecular subgroups. This result prompted us to analyze the co-expression of *PD-1* and *PD-L1* at the mRNA level, the two most promising biomarkers for GC immunotherapy. Studies have shown that patients with mRNA co-expression of *PD-1/PD-L1* were those with better prognosis [36]. However, few GC cases in our cohort presented expression of both markers simultaneously and, interestingly, most were GCLS cases positive for EBV infection. Nevertheless, it has been shown that patients treated with anti-PD-1 therapy respond well even in the absence of PD-L1 expression [37], a fact that may reflect the different co-expression patterns observed in our cohort.

Taking all these observations into account, we further explored the pattern of co-expression of *PD-1* with *Dies1/VISTA* and *CTLA4*. In fact, combination immunotherapies, targeting simultaneously PD-1 and Dies1/VISTA or CTLA4 are being explored as new strategies for the treatment of GC [38,39]. We observed that from the 19 *PD-1* high-expressing GCLS cases, 18 cases also displayed high *Dies1/VISTA* and/or *CTLA4* mRNA expression. This observation suggests that the recognition of GCLS morphological features may contribute, in >70% cases, to the selection of patients who would benefit from a combination immunotherapy, targeting PD-1 and either Dies1/VISTA or CTLA4 (Figure 8). The results herein described raise the hypothesis that Dies1/VISTA and CTLA4 may be the silent PD-1 partners in GC, explaining the good response observed in patients harboring PD-L1-negative tumors, treated with anti-PD-1 therapy [18,19,20,37]. Overall, our results support that most GCLS patients will benefit from anti-PD-1 therapy combined with either anti-Dies1/VISTA or anti-CTLA4 (Figure 8). This novel data is worth further studies. To evaluate, in different GC cohorts, protein expression of multiple immunotherapy targets, besides PD-L1, would be crucial to integrate gene and protein expression data, as well to explore the topographic distribution (i.e., cancer cells versus TME immune cells) of different biomarkers. Of notice, Dies1/VISTA expression has already been assessed in a large GC series, and its expression was mostly detected in >80% of TME immune cells [26].

Our study also revealed that, beyond MSS/MSI-high and EBV infection, the morphological entity GCLS was strongly associated with PD-1 high expression. In fact, ~80% of all GCLS presented high *PD-1* mRNA expression (Figure 8).

Altogether, our data demonstrated that a small transcriptomic panel can separate MSI/EBV− from MSS/EBV+ GC cases, and this may have clinical utility. Also, our analysis demonstrates that EBV+ GCs with GCLS morphological features is the biological subgroup that would more likely respond to immunotherapy, as they present higher *PD-1* expression, the key immunotherapy target in GC, together with *Dies1/VISTA* and *CTLA4*. These observations support the ongoing GC clinical trials and highlight GCLS as a useful feature to stratify patients for targeted immunotherapies.

## 4. Materials and Methods

### 4.1. Case Series

Tissue samples were obtained retrospectively from 46 patients with GC who had undergone gastrectomy as primary treatment at Centro Hospitalar São João (Porto, Portugal). For mRNA extraction, frozen tissue was available from 23 cases, whereas FFPE tissue was used in the remaining 23 cases. The series was enriched with GC cases harboring EBV infection (*n* = 15) and MSI-high status (*n* = 27), whereas the remaining four cases were EBV− and MSS. EBV infection and MSI status were investigated as described below. Histopathological analysis was performed on H&E sections and the tumors were classified as GCLS or CA, based on the abundance of the lymphoid infiltrate.

### 4.2. EBV In Situ Hybridization

The presence of EBV infection was studied by chromogenic *in situ* hybridization (ISH) for EBV− encoded RNA (EBER-ISH, INFORM EBER probe, Ventana Medical Systems, Tucson, AZ, USA). One 3 µm section was processed in the automatic Ventana Benchmark Ultra platform with enzymatic digestion (ISH protease) and the iViewBlue detection kit.

### 4.3. PCR/Fragment Analysis for MSI Status

Genomic DNA was extracted from frozen or FFPE tissues (four sections, each 10 µm thick), using QIAamp DNA Mini Kit (Qiagen, Valencia, CA, USA), in accordance with the manufacturer’s instructions. DNA purity and quantification were assessed using the NanoDrop 2000 UV-Vis spectrophotometer (NanoDrop products, Wilmington, DE, USA). Five mononucleotide markers (BAT-25, BAT-26, NR-24, NR-21 and NR-27) were used as a pentaplex panel to determine MSI status (Multiplex PCR, Qiagen, Valencia, CA, USA). Tumors with instability involving at least two of the five *loci* were classified as MSI.

### 4.4. Gene Expression Profiling by Nanostring nCounter Assay

Total RNA was extracted from frozen or FFPE tissues (four sections, each 10 µm thick), using miRNeasy Mini Kit (Qiagen, Valencia, CA, USA), in accordance with the manufacturer’s instructions. High tumor content of the samples was ensured by morphological evaluation of mirror H&E sections of frozen samples and by microdissection of tumor areas in sections from FFPE blocks. For Nanostring nCounter assay, we used a custom-designed panel comprising 474 genes previously published [23] and additional genes associated with immune response (*CCL22*, *CCR7*, *CD3D*, *CD3E*, *CD3G*, *CD8A*, *CD8B*, *CD19*, *CD20*, *CD45*, *CD68*, *CXCL10*, *CXCL11*, *FOXP3*, *GZMA*, *GZMB*, *IL4*, *IL13*, *PD-1*, *PD-L1*, *TNFA*, *VISTA/Dies1*). Nanostring probe hybridization was performed as a service at Genome Institute of Singapore. Raw counts obtained for each sample were normalized using nSolver software version 3.0 (NanoString Technologies). We performed: (1) background subtraction using eight negative control probes included in the Nanostring CodeSet; (2) positive control normalization using six positive control probes also included in the Nanostring CodeSet; (3) housekeeping normalization using the standard method in the software and five independent housekeeping genes included in the Nanostring CodeSet. Normalized log2-scaled counts were used for to construct heatmaps, dendrograms and to perform PCA described in this study, using the R environment and the packages “ggplot2” and “ggfortify” [40,41,42,43]. Next, each sample was identified concerning its MSS/MSI-high phenotype and/or EBV infection status to perform comparison analysis using also the nSolver software (NanoString Technologies). Calculated ratios and FDR was used to define the set of up/downregulated DE-genes considering the comparison performed: genes with ratio above 1.5 and FDR < 0.05 were classified as differentially expressed upregulated genes; genes with ratio below 0.67 and FDR < 0.05 were classified as differentially expressed downregulated genes.

### 4.5. PD-L1 Immunohistochemistry

Staining for PD-L1 was performed in FFPE 3 µm sections with a rabbit monoclonal antibody (clone E1L3N, 1:1000; Cell Signaling Technology) on the automatic Ventana Benchmark Ultra platform, using the OptiView Universal DAB detection kit and the OptiView Amplification kit from the same manufacturer. PD-L1 immunoexpression was evaluated semi-quantitatively for tumor epithelial and stromal immune cells, according to the immunoreactivity scoring system (IRS) described by Boger et al. [44]. PD-L1 expression in tumor epithelial cells was dichotomized as positive (detected) or negative (absent) by an immunoreactivity score (IRS) of 2. PD-L1 expression in immune cells of the TME was defined as low (1–5% of positive cells), intermediate (6–20% of positive cells) or high (>20% of positive cells).

### 4.6. Functional Annotation and Statistical Analysis

Functional annotation was performed using the online tool DAVID 6.7 [24,25]. In particular, we have used the option ‘Functional Annotation Clustering’ using always the stringency ‘high’ and the option ‘Functional Annotation Chart’. Selected clusters and/or biological terms were considered enriched and reported in this study if presenting an FDR < 0.05. Normalized log2-scaled counts for the genes *CTLA4*, *PD-1*, *VISTA/Dies1* and *PD-L1* was collected from nSolver software (NanoString Technologies), as previously described. Boxplots were plotted using R [40] and represented *p*-values derived from a Wilcoxon test (Mann-Whitney) also performed using R and all samples (including outliers). After building a contingency table for the number of cases in each detailed condition, a Fisher’s exact test was performed using R. This test was selected rather than the chi-square test, due to the low number of samples available in our cohort.

## 5. Conclusions

In this study, we have shown that the expression profile of GC cases for the assessed 499 genes was strongly correlated with the established molecular subtypes currently used for GC molecular stratification. Altogether, our results have clearly associated: (1) MSI-high/EBV− GC cases with mitosis and cell cycle biological terms; (2) MSS/EBV+ GC cases with immune response mediated by T-cells. Importantly, we have also shown that the MSI status is a much more relevant molecular classifier than EBV infection. We have also revealed that *PD-L1* and *PD-1* have opposite mRNA expression patterns in GC, in correlation with MSI phenotype, EBV status and prominent immune infiltrate, as revealed by the GCLS morphological feature, a highly relevant finding as both genes are nowadays actively pursued as targets for immunotherapy in GC. Moreover, our study has shown that Dies1/VISTA and CTLA4 are highly expressed in the majority of EBV+ and GCLS cases, strengthening the relevance of clinical trials using antibodies raised against these two proteins in combination with the promising anti-PD-1 therapy.

## Figures and Tables

**Table 1:** Clustering results using a *k*-means approach. Represented is the number of MSI-high/MSS and EBV+/EBV− cases obtained in each cluster for each *k* value.

Value of *k*	Cluster ID	MSI Status		EBV Infection	
		MSI-High (*n* = 27)	MSS (*n* = 19)	EBV+ (*n* = 15)	EBV− (*n* = 31)
2	I	25	1	1	26
	II	2	18	14	6
3	I	27	0	0	27
	II	0	2	2	0
	III	0	17	13	4
4	I	13	0	0	13
	II	14	0	0	14
	III	0	17	13	4
	IV	0	2	2	0
5	I	0	12	9	3
	II	12	0	0	12
	III	0	2	2	0
	IV	12	0	0	12
	V	3	5	4	4

**Table 2:** FDR-ranked top 10 biological terms significantly enriched in the set of 193 DE-genes when comparing the expression profile for 499 genes between MSS and MSI-high cases.

Biological Term	Count	FDR
Signal Peptide	73	1.24 × 10^−11^
Disulfide bond	64	1.05 × 10^−09^
Secreted	44	2.67 × 10^−7^
GO:0042127~regulation of cell proliferation	31	1.77 × 10^−6^
GO:0006935~chemotaxis	15	3.16 × 10^−6^
GO:0042330~taxis	15	3.16 × 10^−6^
IPR001811:Small chemokine, interleukin-8-like	9	8.69 × 10^−6^
GO:0007626~locomotory behavior	18	1.08 × 10^−5^
GO:0045321~leukocyte activation	17	1.26 × 10^−5^
GO:0008009~chemokine activity	9	2.10 × 10^−5^

Count: number of DE-genes associated with a given biological term; FDR: False Discovery Rate.

**Table 3:** Functional annotation clustering results for DE-genes separated according to the expression profile in MSS vs. MSI-high cases. Three clusters of associated biological terms were detected for upregulated DE-genes, while only one was detected for downregulated DE-genes. Notice that some terms among clusters are not significantly enriched (FDR > 0.05).

Cluster ID	Term	Count	FDR
Upregulated DE-genes in MSI-high vs. MSS cases (*n* = 55)			
Cluster 1: ES = 9.5	GO:0022402~cell cycle process	17	1.46 × 10^−8^
	GO:0000279~M phase	14	3.24 × 10^−8^
	GO:0000280~nuclear division	12	1.17 × 10^−7^
	GO:0007067~mitosis	12	1.17 × 10^−7^
	GO:0000278~mitotic cell cycle	14	1.40 × 10^−7^
	GO:0000087~M phase of mitotic cell cycle	12	1.43 × 10^−7^
	GO:0048285~organelle fission	12	1.81 × 10^−7^
	GO:0022403~cell cycle phase	14	5.62 × 10^−7^
	mitosis	10	1.09 × 10^−6^
	GO:0007049~cell cycle	17	1.62 × 10^−6^
	cell division	11	1.48 × 10^−6^
	cell cycle	12	2.50 × 10^−5^
	GO:0051301~cell division	11	4.02 × 10^−5^
Cluster 2: ES = 4.3	GO:0005819~spindle	9	2.00 × 10^−5^
	GO:0015630~microtubule cytoskeleton	10	5.42 × 10^−2^
	GO:0044430~cytoskeletal part	10	3.05
	GO:0005856~cytoskeleton	12	3.72
Cluster 3: ES = 3.7	GO:0007052~mitotic spindle organization	4	2.13 × 10^−2^
	GO:0007051~spindle organization	4	6.18 × 10^−1^
	GO:0000226~microtubule cytoskeleton organization	5	1.92
Downregulated DE-genes in MSI-high vs. MSS cases (*n* = 138)			
Cluster 1: ES = 16.0	disulfide bond	57	1.67 × 10^−13^
	signal peptide	61	1.67 × 10^−13^
	signal	61	1.44 × 10^−13^
	disulfide bond	57	5.66 × 10^−13^
Cluster 2: ES = 9.7	GO:0007626~locomotory behavior	17	2.98 × 10^−7^
	GO:0042330~taxis	14	3.03 × 10^−7^
	GO:0006935~chemotaxis	14	3.03 × 10^−7^
Cluster 3: ES = 8.2	GO:0046649~lymphocyte activation	14	4.51 × 10^−6^
	GO:0045321~leukocyte activation	15	5.12 × 10^−6^
	GO:0001775~cell activation	15	4.50 × 10^−5^
Cluster 4: ES = 5.4	IPR001811: Small chemokine, interleukin-8-like	8	1.75 × 10^−5^
	GO:0008009~chemokine activity	8	5.02 × 10^−5^
	GO:0042379~chemokine receptor binding	8	7.91 × 10^−5^
	SM00199:SCY	8	1.88 × 10^−4^
	cytokine	9	2.47 × 10^−2^
	109.Chemokine_families	8	6.13 × 10^−2^
	GO:0005125~cytokine activity	9	1.18 × 10^−1^
	hsa04062:Chemokine signaling pathway	10	4.13 × 10^−1^
	hsa04060:Cytokine-cytokine receptor interaction	11	1.19
Cluster 5: ES = 5.1	GO:0009719~response to endogenous stimulus	15	2.99 × 10^−3^
	GO:0009725~response to hormone stimulus	14	5.54 × 10^−3^
	GO:0010033~response to organic substance	17	1.38 × 10^−1^
Cluster 6: ES = 4.9	GO:0043067~regulation of programmed cell death	20	1.27 × 10^−2^
	GO:0010941~regulation of cell death	20	1.34 × 10^−2^
	GO:0042981~regulation of apoptosis	19	4.16 × 10^−2^
Cluster 7: ES = 4.9	GO:0016477~cell migration	12	1.10 × 10^−2^
	GO:0006928~cell motion	15	1.88 × 10^−2^
	GO:0048870~cell motility	12	2.99 × 10^−2^
	GO:0051674~localization of cell	12	2.99 × 10^−2^
Cluster 8: ES = 4.3	GO:0030247~polysaccharide binding	9	2.18 × 10^−2^
	GO:0001871~pattern binding	9	2.18 × 10^−2^
	GO:0005539~glycosaminoglycan binding	8	9.63 × 10^−2^
	GO:0030246~carbohydrate binding	11	3.40 × 10^−1^

ES: enrichment score per cluster calculated by DAVID. FDR: False Discovery Rate.

**Table 4:** FDR-ranked top 10 biological terms significantly enriched in the set of 142 DE-genes when comparing the expression profile for 499 genes between EBV+ and EBV− GC cases.

Biological Term	Count	FDR
signal peptide	54	1.38 × 10^−8^
IPR001811:Small chemokine, interleukin-8-like	10	1.40 × 10^−8^
GO:0042330~taxis	15	3.13 × 10^−8^
GO:0006935~chemotaxis	15	3.13 × 10^−8^
disulfide bond	49	5.16 × 10^−8^
GO:0008009~chemokine activity	10	6.98 × 10^−8^
GO:0042379~chemokine receptor binding	10	1.28 × 10^−7^
SM00199:SCY	10	1.99 × 10^−7^
GO:0007626~locomotory behavior	17	4.65 × 10^−7^
GO:0006955~immune response	25	5.25 × 10^−7^

Count: number of DE-genes associated with a given biological term; FDR: False Discovery Rate.

**Table 5:** Functional annotation clustering results for the 141 DE-genes upregulated in EBV+ cases. Six clusters of associated biological terms were detected for upregulated DE-genes and two clusters for downregulated DE-genes. Notice that some terms among clusters are not significantly enriched (FDR > 0.05).

Cluster ID	Term	Count	FDR
Upregulated DE-genes in EBV+ vs. EBV− cases (*n* = 105)			
Cluster 1: ES = 12.2	disulfide bond	44	1.36 × 10^−10^
	disulfide bond	44	3.56 × 10^−10^
	signal	45	2.94 × 10^−9^
	signal peptide	45	4.07 × 10^−9^
Cluster 2: ES = 10.3	GO:0042330~taxis	14	5.99 × 10^−9^
	GO:0006935~chemotaxis	14	5.99 × 10^−9^
	GO:0007626~locomotory behavior	16	3.41 × 10^−8^
	GO:0007610~behavior	16	5.57 × 10^−5^
Cluster 3: ES = 10	GO:0045321~leukocyte activation	15	8.26 × 10^−8^
	GO:0046649~lymphocyte activation	14	9.54 × 10^−8^
	GO:0042110~T-cell activation	12	1.43 × 10^−7^
	GO:0001775~cell activation	15	7.88 × 10^−7^
Cluster 4: ES = 7.4	IPR001811:Small chemokine, interleukin-8-like	9	3.94 × 10^−8^
	GO:0008009~chemokine activity	9	1.52 × 10^−7^
	GO:0042379~chemokine receptor binding	9	2.60 × 10^−7^
	SM00199:SCY	9	4.73 × 10^−7^
	cytokine	10	2.20 × 10^−4^
	GO:0005125~cytokine activity	10	1.39 × 10^−3^
	hsa04062:Chemokine signaling pathway	11	1.09 × 10^−2^
	109.Chemokine_families	9	1.22 × 10^−2^
	hsa04060:Cytokine-cytokine receptor interaction	12	3.67 × 10^−2^
Cluster 5: ES = 5.2	GO:0002520~immune system development	11	4.50 × 10^−3^
	GO:0030097~hemopoiesis	10	9.75 × 10^−3^
	GO:0002521~leukocyte differentiation	8	1.28 × 10^−2^
	GO:0048534~hemopoietic or lymphoid organ development	10	2.13 × 10^−2^
Cluster 6: ES = 5.2	GO:0030217~T-cell differentiation	7	2.48 × 10^−3^
	GO:0002521~leukocyte differentiation	8	1.28 × 10^−2^
	GO:0030098~lymphocyte differentiation	7	3.64 × 10^−2^
Downregulated DE-genes in EBV+ vs. EBV− cases (*n* = 137)			
Cluster 1: ES = 5.2	GO:0000279~M phase	8	5.32 × 10^−3^
	cell division	7	4.85 × 10^−3^
	GO:0007067~mitosis	7	7.12 × 10^−3^
	GO:0000280~nuclear division	7	7.12 × 10^−3^
	GO:0000087~M phase of mitotic cell cycle	7	7.90 × 10^−3^
	GO:0048285~organelle fission	7	8.97 × 10^−3^
	cell cycle	8	9.93 × 10^−3^
	mitosis	6	1.38 × 10^−2^
	GO:0051301~cell division	7	3.79 × 10^−2^
Cluster 2: ES = 5	GO:0022402~cell cycle process	11	1.46 × 10^−4^
	GO:0005819~spindle	7	5.61 × 10^−4^
	GO:0000278~mitotic cell cycle	9	8.25 × 10^−4^
	GO:0022403~cell cycle phase	9	1.93 × 10^−3^
	GO:0007049~cell cycle	11	2.74 × 10^−3^
	GO:0015630~microtubule cytoskeleton	8	1.20 × 10^−1^
	GO:0044430~cytoskeletal part	8	3.21
	GO:0005856~cytoskeleton	9	6.62

ES: enrichment score per cluster calculated by DAVID; FDR: False Discovery Rate.

**Table 6:** FDR-ranked top 10 biological terms significantly enriched in the set of 166 DE-genes when comparing the expression profile for 499 genes between MSS vs. MSI-high and EBV+ vs. EBV− GC cases.

Biological Term	Count	FDR
IPR001811:Small chemokine, interleukin-8-like	10	6.17 × 10^−8^
signal peptide	58	1.97 × 10^−7^
GO:0008009~chemokine activity	10	2.18 × 10^−7^
GO:0006935~chemotaxis	15	3.16 × 10^−7^
GO:0042330~taxis	15	3.16 × 10^−7^
disulfide bond	53	3.22 × 10^−7^
GO:0042379~chemokine receptor binding	10	3.97 × 10^−7^
SM00199:SCY	10	8.05 × 10^−7^
disulfide bond	53	8.56 × 10^−7^
GO:0045321~leukocyte activation	17	9.73 × 10^−7^

Count: number of DE-genes associated with a given biological term; FDR: False Discovery Rate.

**Table 7:** Functional annotation clustering results for the 166 DE-genes derived from the comparison of MSS/EBV+ cases vs. MSI-high/EBV− cases. Notice that some terms among clusters are not significantly enriched (FDR > 0.05).

Cluster ID	Term	Count	FDR
Upregulated DE-genes in MSS/EBV+ cases (*n* = 117)			
Cluster 1: ES = 12.5	disulfide bond	47	1.12 × 10^−10^
	disulfide bond	47	3.06 × 10^−10^
	signal	49	7.15 × 10^−10^
	signal peptide	49	1.03 × 10^−9^
Cluster 2: ES = 9.6	GO:0042330~taxis	14	2.32 × 10^−8^
	GO:0006935~chemotaxis	14	2.32 × 10^−8^
	GO:0007626~locomotory behavior	16	1.58 × 10^−7^
	GO:0007610~behavior	16	2.31 × 10^−4^
Cluster 3: ES = 8.3	GO:0045321~leukocyte activation	14	4.01 × 10^−6^
	GO:0046649~lymphocyte activation	13	4.72 × 10^−6^
	GO:0042110~T-cell activation	11	7.75 × 10^−6^
	GO:0001775~cell activation	14	3.11 × 10^−5^
Cluster 4: ES = 6.8	IPR001811:Small chemokine, interleukin-8-like	9	9.74 × 10^−8^
	GO:0008009~chemokine activity	9	3.30 × 10^−7^
	GO:0042379~chemokine receptor binding	9	5.62 × 10^−7^
	SM00199:SCY	9	1.34 × 10^−6^
	cytokine	9	5.94 × 10^−3^
	109.Chemokine_families	9	4.48 × 10^−3^
	hsa04062:Chemokine signaling pathway	11	1.87 × 10^−2^
	GO:0005125~cytokine activity	9	2.77 × 10^−2^
	hsa04060:Cytokine-cytokine receptor interaction	11	3.23 × 10^−1^
Cluster 5: ES = 4.6	GO:0001725~stress fiber	5	2.38 × 10^−2^
	GO:0032432~actin filament bundle	5	3.31 × 10^−2^
	GO:0042641~actomyosin	5	3.87 × 10^−2^
Downregulated DE-genes in MSS/EBV+ cases (*n* = 49)			
Cluster 1: ES = 9.9	GO:0000278~mitotic cell cycle	14	2.51 × 10^−8^
	GO:0000280~nuclear division	12	2.79 × 10^−8^
	GO:0007067~mitosis	12	2.79 × 10^−8^
	GO:0000087~M phase of mitotic cell cycle	12	3.39 × 10^−8^
	GO:0048285~organelle fission	12	4.30 × 10^−8^
	GO:0022403~cell cycle phase	14	1.02 × 10^−7^
	GO:0000279~M phase	13	1.15 × 10^−7^
	mitosis	10	3.25 × 10^−7^
	cell division	11	3.84 × 10^−7^
	cell cycle	12	5.83 × 10^−6^
	GO:0051301~cell division	11	1.15 × 10^−5^
Cluster 2: ES = 3.5	GO:0005819~spindle	7	4.12 × 10^−3^
	GO:0015630~microtubule cytoskeleton	9	1.60 × 10^−1^
	GO:0005856~cytoskeleton	11	5.27
	GO:0044430~cytoskeletal part	9	5.64

ES: enrichment score per cluster calculated by DAVID; FDR: False Discovery Rate.

**Table 8:** Contingency table for the number of MSS/EBV− or MSS/EBV+ or MSI/EBV− cases with morphological features of GCLS or CA for the four *PD-L1/PD-1* mRNA expression scenarios.

mRNA Expression		MSS/EBV− (*n* = 4)		MSS/EBV+ (*n* = 15)		MSI/EBV− (*n* = 27)	
*PD-L1*	*PD-1*	CA (*n* = 0)	GCLS (*n* = 4)	CA (*n* = 0)	GCLS (*n* = 15)	CA (*n* = 21)	GCLS (*n* = 6)
↗	↙	0	0	0	0	10	2
↙	↗	0	3	0	7	0	2
↙	↙	0	1	0	2	10	1
↗	↗	0	0	0	6	1	1
Fisher’s Exact Test: *p* = 3.71 × 10^−6^							

## Abbreviations

CA	Conventional-type Adenocarcinoma
DE	Differentially Expressed
EBV	Epstein-Barr Virus
ES	Enrichment Score
FDR	False Discovery Rate
GC	Gastric Cancer
GCLS	Gastric Cancer with Lymphoid Stroma
IFNγ	Interferon gamma
IHC	Immunohistochemistry
MSI-high	Microsatellite unstable
MSS	Microsatellite stable
PD-1	Program Death 1
PD-L1	Program Death Ligand 1
TCGA	The Cancer Genome Atlas
TME	Tumor Microenvironment
